# Supplementary material for: Content-rich biological network constructed by mining PubMed abstracts
Source: BMC Bioinformatics. 2004 Oct 8;5:147. doi: 10.1186/1471-2105-5-147 (PMC528731; doi:10.1186/1471-2105-5-147)
Supplement: Additional File 2 — The original results of the above study (non-essential files are deleted to keep the file size under the limit set by BMC bioinformatics). [file 1471-2105-5-147-S2.bz2 › chilibotAdditionalFile2/dip10/239ID9054408E781/html/X_GTF2B.html]

 


 **X** and **GTF2B** 
  
Found 595 abstracts in PubMed, retrieved 10.  
 

 What does Google say? 
 PDF only 
| .edu only 

---

**Interactive relationship** (e.g. stimulation, inhibition, etc)

**Neutral relationship**- From our analysis, the K off 0, the single molecular binding energy E b, and the transition state  **x**  b, were extracted from the data, and estimated to be 1.53s.64x10 20 J and 1.03A for the RGD alpha IIb  [ **GTF2B** ]  beta 3 system, and 47.58s.67x10 2.09A for the HHLGGAKQAGDV alpha IIb  [ **GTF2B** ]  beta 3 system, respectively.  Ref: 12801687 Ultramicroscopy, 2003

**Non-interactive relationship** (e.g. studied together, co-existance, homology, etc.)

- In particular, a high level of collagen type IIB  [ **GTF2B** ]  expression was associated with three dimensional culture conditions, and the HYAFF 11 material was the most supportive for human collagen type  **X**  mRNA expression.  Ref: 12763454 Biomaterials, 2003
- The slope of the force frequency curve was lower in patients with ischemia compared with those without group IIA = 3.5 4.2  **x**  10 2 vs group IIB  [ **GTF2B** ]  = 11.4 9.5  **x**  10 2.  Ref: 12778025 J Am Soc Echocardiogr, 2003
